# Targeting CSF1R Alone or in Combination with PD1 in Experimental Glioma

**DOI:** 10.3390/cancers13102400

**Published:** 2021-05-15

**Authors:** Justyna M. Przystal, Hannes Becker, Denis Canjuga, Foteini Tsiami, Nicole Anderle, Anna-Lena Keller, Anja Pohl, Carola H. Ries, Martina Schmittnaegel, Nataliya Korinetska, Marilin Koch, Jens Schittenhelm, Marcos Tatagiba, Christian Schmees, Susanne C. Beck, Ghazaleh Tabatabai

**Affiliations:** 1 Department of Neurology & Interdisciplinary Neuro-Oncology, Hertie Institute for Clinical Brain Research, Center for Neuro-Oncology, Comprehensive Cancer Center, University Hospital Tübingen, Eberhard Karls University Tübingen, 72076 Tübingen, Germany; przystal@kispi.uzh.ch (J.M.P.); hannes.becker@student.uni-tuebingen.de (H.B.); denis.canjuga@gmx.net (D.C.); foteini.tsiami@student.uni-tuebingen.de (F.T.); nataliya.korinetska@med.uni-tuebingen.de (N.K.); Marilin.Koch@med.uni-tuebingen.de (M.K.); marcos.tatagiba@med.uni-tuebingen.de (M.T.); su.beck@uni-tuebingen.de (S.C.B.); 2 German Translational Cancer Consortium (DKTK), DKFZ Partner Site Tübingen, 72076 Tübingen, Germany; jens.schittenhelm@med.uni-tuebingen.de; 3 NMI, Natural and Medical Sciences Institute, University of Tübingen, 72770 Reutlingen, Germany; Nicole.Anderle@nmi.de (N.A.); anna-lena.keller@nmi.de (A.-L.K.); anja.pohl@nmi.de (A.P.); Christian.Schmees@nmi.de (C.S.); 4 Roche Innovation Center Munich, Oncology Division, Roche Pharmaceutical Research and Early Development, 82377 Penzberg, Germany; riescarola@gmx.de (C.H.R.); martina.schmittnaegel@roche.com (M.S.); 5 Institute for Neuropathology, University Hospital Tübingen, 72076 Tübingen, Germany; 6 Department of Neurosurgery, University Hospital Tübingen, Eberhard Karls University Tübingen, 72076 Tübingen, Germany; 7 Cluster of Excellence iFIT (EXC 2180) “Image Guided and Functionally Instructed Tumor Therapies”, Eberhard Karls University Tübingen, 72076 Tübingen, Germany

**Keywords:** CSF1R, PD1, glioblastoma, sequential therapy, immunotherapy

## Abstract

**Simple Summary:**

Glioblastomas are incurable tumors of the central nervous system. Currently, treatment strategies combine neurosurgical intervention, radiation therapy, and chemotherapy. Yet, clinical experience shows that tumors acquire escape mechanisms. Furthermore, the tumor-associated microenvironment, including macrophages expressing the receptor CSF1R, promote and nourish tumor cells. The so-called PD1/PDL1 axis is a major reason why tumors can grow with a “magic hat”; i.e., unrecognized from the immune system. The aim of our study was to assess treatment strategies that target macrophages in the microenvironment by blocking CSF1R alone or in combination with PD1 blockade. Using an immune competent mouse model and an ex vivo microtumor model using freshly resected glioblastoma material, we observed prolonged survival of treated mice and an improved “attack” of the immune system. We conclude that targeting CSF1R is a promising strategy that should be explored in clinical trials, potentially in combination with PD1 blockade.

**Abstract:**

Glioblastoma is an aggressive primary tumor of the central nervous system. Targeting the immunosuppressive glioblastoma-associated microenvironment is an interesting therapeutic approach. Tumor-associated macrophages represent an abundant population of tumor-infiltrating host cells with tumor-promoting features. The colony stimulating factor-1/ colony stimulating factor-1 receptor (CSF-1/CSF1R) axis plays an important role for macrophage differentiation and survival. We thus aimed at investigating the antiglioma activity of CSF1R inhibition alone or in combination with blockade of programmed death (PD) 1. We investigated combination treatments of anti-CSF1R alone or in combination with anti-PD1 antibodies in an orthotopic syngeneic glioma mouse model, evaluated post-treatment effects and assessed treatment-induced cytotoxicity in a coculture model of patient-derived microtumors (PDM) and autologous tumor-infiltrating lymphocytes (TILs) ex vivo. Anti-CSF1R monotherapy increased the latency until the onset of neurological symptoms. Combinations of anti-CSF1R and anti-PD1 antibodies led to longterm survivors in vivo. Furthermore, we observed treatment-induced cytotoxicity of combined anti-CSF1R and anti-PD1 treatment in the PDM/TILs cocultures ex vivo. Our results identify CSF1R as a promising therapeutic target for glioblastoma, potentially in combination with PD1 inhibition.

## 1. Introduction

Glioblastoma is an incurable aggressive primary brain tumor. The median overall survival is still in the range of 1.5 years despite multimodal therapy even in selected clinical trial population [1,2,3,4,5], and 5-year survival rates are only approximately 5% [6]. Glioblastomas efficiently reprogram their microenvironment towards an immunosuppressive milieu [7] by altered surface molecule expressions, e.g., human leucocyte antigen (HLA)-E and lectin-like transcript-1 (LLT-1) [8,9]. Moreover, upregulated signal transducer and activator of transcription 3 (STAT3) induces the production of immunosuppressive cytokines like transforming growth factor (TGF)-beta and interleukin (IL)-6 [10,11]. Consequently, immunotherapeutic strategies aimed at overcoming this glioblastoma-associated immunosuppressive signature are considered promising. Various approaches are currently in clinical development, e.g., peptide vaccination, cellular therapies, and immune checkpoint blockade. Immune checkpoint blockade with antibodies targeting the programmed cell death (PD)1 led to promising results in several metastatic cancers [12]. They act by interfering with the interaction between PD1 and the respective ligands and thereby disrupting the inhibitory effects on T cell-mediated immune reaction [13]. However, PD1 inhibition did not led to the same clinical outcome in glioblastoma. In progressive glioblastoma, nivolumab was not superior compared with bevacizumab (NCT02017717). Investigations of the efficacy of nivolumab in newly diagnosed glioblastoma are currently ongoing (NCT02617589, NCT02667587). Postoperative treatment with PD1 antibody and radiation therapy in *O*^6^-methylguanine DNA methyltransferase (MGMT)-unmethylated newly diagnosed glioblastoma did not improve overall survival compared with radiation therapy and temozolomide (NCT02617589).

A potential strategy for enhancing the efficacy of PD1 in glioblastoma might be the design of rational combination therapies. In melanoma, mining of publicly available transcriptomic data sets indicated a coenrichment of CD8^+^ T cells with colony stimulating factor (CSF)1 and other macrophage-specific markers, which were associated with nonresponsiveness to PD1 blockade [14]. In human gliomas, expression of CSF1 is present in glial fibrillary acidic protein (GFAP)-positive cells [15]. Cultured glioblastoma sphere-forming cells release CSF1 [16]. Moreover, CSF1 has an oncogenic role in gliomagenesis [17]. Yet, a Phase II study investigating the compound PLX3397, an oral small molecule inhibitor targeting CSF1R and KIT, in 37 patients, suggested that the compound is well-tolerated, but monotherapy has no efficacy [18]. The inhibition of CSF1R in a preclinical study using the RCAS-hPDGF-B/Nestin-Tv-a; Ink4a/Arf^−/−^ model led to prolonged overall survival [19]. Moreover, microenvironmental alterations by CSF1R blockade rendered tumor cells susceptible to receptor tyrosine kinase inhibitors dovitinib and vatalinib in preclinical studies [20].

Based on these facts, we hypothesized that CSF1R blockade might be a promising therapeutic strategy, either as monotherapy or in combination with PD1 inhibition [21].

## 2. Results

### 2.1. Expression of CSF1R, CD204, CD163, PD1, and PD-L1 in Primary and Progressive Glioblastoma

**Figure 1:**
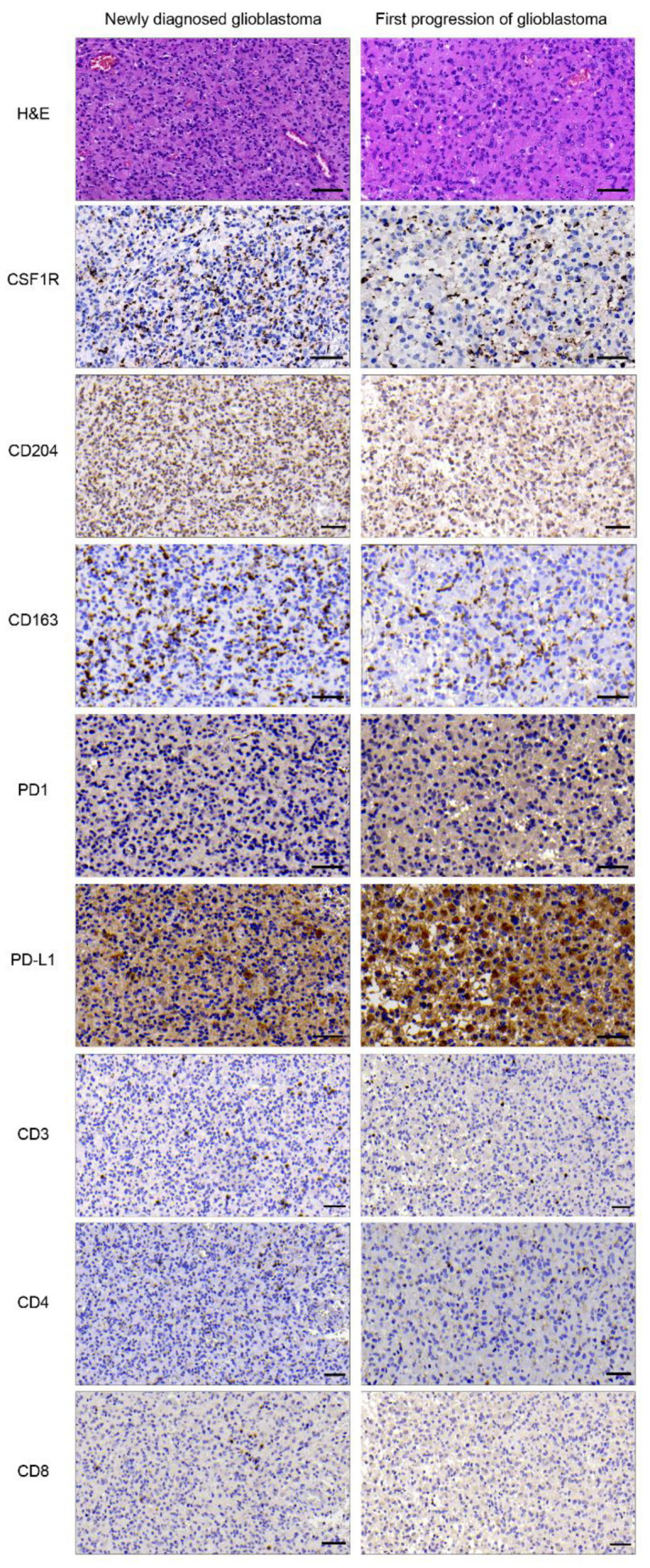
CSF1R and PD1 are present in primary and progressive glioblastoma. Representative tumor areas from matched pairs of newly diagnosed and progressive glioblastoma. H&E staining (top row) and immunohistochemical staining of CSF1R (*n* = 28), CD204 (*n* = 27), CD163 (*n* = 31), PD1 (*n* = 30), PD-L1 (*n* = 31), CD3 (*n* = 28), CD4 (*n* = 30), and CD8 (*n* = 28). Scale bars 50 µm.

We investigated paired human glioblastoma samples from primary and subsequent progressive disease for the presence of CSF1R, CD204, CD163, PD1, PD-L1, CD3, CD4, and CD8 (as illustrated in Figure 1). Six patients received radiotherapy only between first diagnosis and progression, and 28 of 34 (82.4%) patients were treated with radiation therapy and concomitant and adjuvant temozolomide [22]. For the analysis of the immunohistochemical stainings, expression levels of tissue-dependent markers were assessed. The expression of all markers was observed in most cases with usually low to intermediate levels (as illustrated in Supplementary Table S2). We used the established immunoreactive score (IRS) to link semiquantitative staining frequency and intensity pattern. IRS calculations demonstrate the presence and strong staining signal particularly of CD204 and CSF1R in both primary and corresponding progressive tissue (as illustrated in Supplementary Figure S1). Highest mean IRS values were observed for tumor-associated macrophages marker CD204 (mean_CD204_: 7.16) and T cell marker CD4 (mean_CD4_: 5.74). Additionally, the treatment target CSF1R was consistently present (mean_CSF1R_: 4.57), and PD1 IRS were rather less seen in this cohort (mean_PD1_: 0.29). Furthermore, the frequency of tumor-infiltrating lymphocytes (TILs) and tumor-associated macrophages (TAMs) was stable in progressive compared with that of newly diagnosed tumor tissue.

Intrapatient expression patterns reveal rather stable expression of tumoral microenvironmental markers (as illustrated in Supplementary Table S3). As an example, 57.1% and 60% of the samples show the same expression levels for CSF1R and PD1 in primary and recurrent tumor situations (as illustrated in Supplementary Table S3).

Next, we performed correlation analysis of tissue-based parameters. Potential correlation of TAMs and TILs markers were of particular interest to link presence of both compartments inside the tumor microenvironment in newly diagnosed and progressive glioblastoma tissue (as illustrated in Supplementary Figure S2). Strongest positive linear correlation was found between CD204 and PD-L1 (correlation coefficient r_recurr_ = 0.843, *p*_recurr_ < 0.0001). PD-L1 expression revealed intermediate to strong association with CD163 expression (r_prim_ = 0.459, *p*_prim_ < 0.006; r_recurr_ = 0.643, *p*_recurr_ < 0.0001) and CSF1R (r_prim_ = 0.492, *p*_prim._ < 0.005). CSF1R showed moderate correlation with TAM-marker CD204, too (r_prim_ = 0,381, *p* < 0.003) We detected a correlation between PD1 and CD4 (r_prim_ = 0.323, *p*_prim._ < 0.047). CD163 revealed intermediate association with CSF1R (r_prim_ = 0.492, *p*_prim_ < 0.005), as well as with CD4 and CD8 (r_prim_ = 0.373, *p*_prim._ < 0.025).

T cell specific markers showed either strong or intermediate positive linear correlation; for instance, general T cell marker CD3 correlated with CD4 and CD8 (r_prim_ = 0.548, *p*_prim._ < 0.001; r_recurr_ = 0.569; *p*_recurr_ < 0.003/0.039) (as illustrated in Supplementary Figure S2). The remaining subgroups and tissue-dependent markers did not reveal significant effect sizes and correlations.

Taken together, our stainings detected CSF1R and markers for TAM and TILs in newly diagnosed and corresponding progressive glioblastoma samples. Our correlation analysis mainly revealed an association between immunosuppressive signature (PD1 and PD-L1) and TAMs/TILs markers. Our data further suggest that CSF1R and PD1 stainings are comparable (as illustrated in Supplementary Table S2) in newly diagnosed and corresponding progressive disease.

### 2.2. Monotherapies with PD1 Antibody and CSF1R Antibody Prolong the Latency until the Onset of Neurological Symptoms In Vivo and Lead to an Altered Immune Signature in the Microenvironment

**Figure 2:**
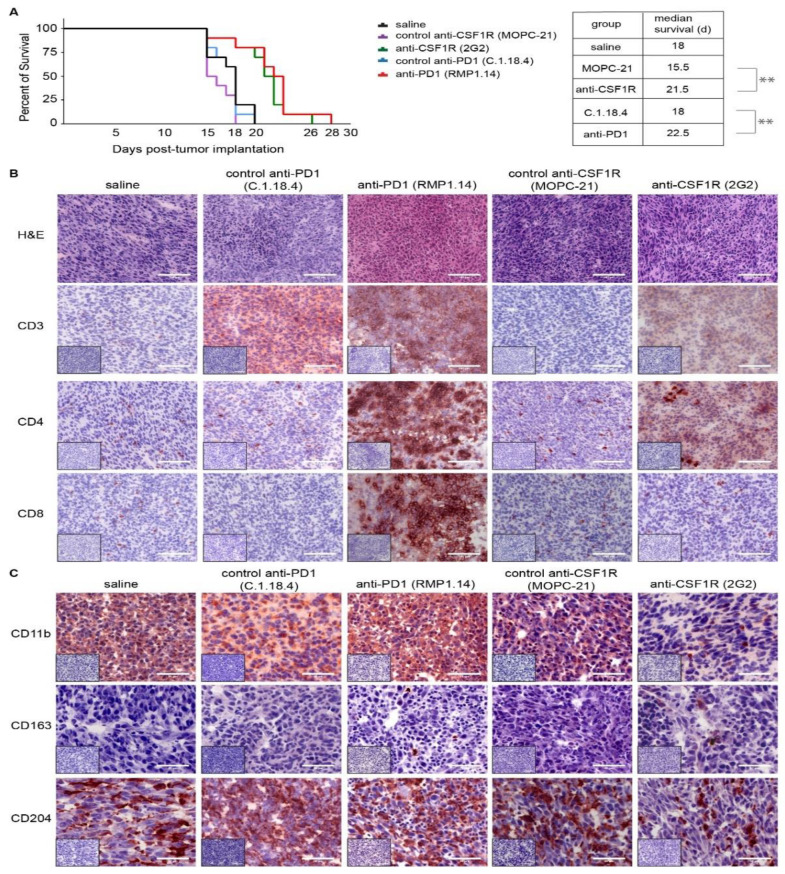
Monotherapies with PD1 and CSF1R blockade in experimental syngeneic SMA-560 glioma in vivo. (**A**): Kaplan–Meier plot showing symptom-free survival. Experimental groups (*n* = 10 in each group) include control treatment (saline), anti-CSF1R (2G2) antibody, anti-PD1 (RPM1.14) antibody, and respective control antibodies. Treatments started on day 14 post-tumor implantation. Tukey–Kramer post hoc test was used after performing Log-rank (Mantel–Cox) test (*p* < 0.001). ** *p* < 0.001 Survival time depicted in Kaplan–Meier plot refers to experimental endpoint as described in detail in material/methods section and in Supplementary Table S1. (**B**,**C**): Immunohistochemical analysis in post-treatment SMA-560 gliomas of one representative animal per group (*n* = 1). Small inserts show staining control without application of primary antibody. (scale bars in (**B**): 100 μm; scale bars in (**C**): 50 μm).

We first investigated the efficacy of monotherapies with PD1 or CSF1R antibody in a syngeneic mouse model. We implanted SMA-560 tumor cells into the right striatum of VM/Dk mice (day 0) and started the treatment on day 14 with the anti-CSF1R antibody 2G2, or the anti-PD1 antibody RMP1.14 or the respective control antibodies. The median survival time in the control group was 18 days, in the anti-CSF1R group 22 days, and in the anti-PD1 group 23 days (as illustrated in Figure 2A). The survival time refers to the experimental endpoint as outlined in material/methods.

We performed immunohistochemical analyses on post-treatment brains of each experimental group and investigated infiltrations of tumor tissues by T cells and macrophages/microglia using CD3, CD4, CD8 (as illustrated in Figure 2B), CD11b, CD163, C204 (as illustrated in Figure 2C), and PD1 and PD-L1 (as illustrated in Supplementary Figure S4). In the control (saline treatment) group, CD3, CD4, CD8 stainings revealed decreased staining distribution with only few single positive cells (as illustrated in Figure 2B, first column). Of note, CD11b and CD204 stainings showed strong signals (as illustrated in Figure 2C, first column). This pattern was similar in the control antibody-treated tissues (as illustrated in Figure 2A,B, second and fourth column). In contrast, the tumor tissue after a treatment with the anti-PD1 antibody showed strong stainings for CD3-, CD4-, and CD8-positive cells (as illustrated in Figure 2B, third column) and unaltered strong CD204 positivity (as illustrated in Figure 2C, third column). The treatment with the anti-CSF1R antibody led to an increase of CD4-positive cells (as illustrated in Figure 2A, last column) and a reduction of CD11b- and CD204-positive cells (as illustrated in Figure 2C, last column).

### 2.3. Combinations of Anti-CSF1R and Anti-PD1 Antibodies Lead to Longer Term Surviving Animals In Vivo If Applied Simultaneously or Sequentially, but Only If PD1 Blockade Follows CSF-1R Blockade

**Figure 3:**
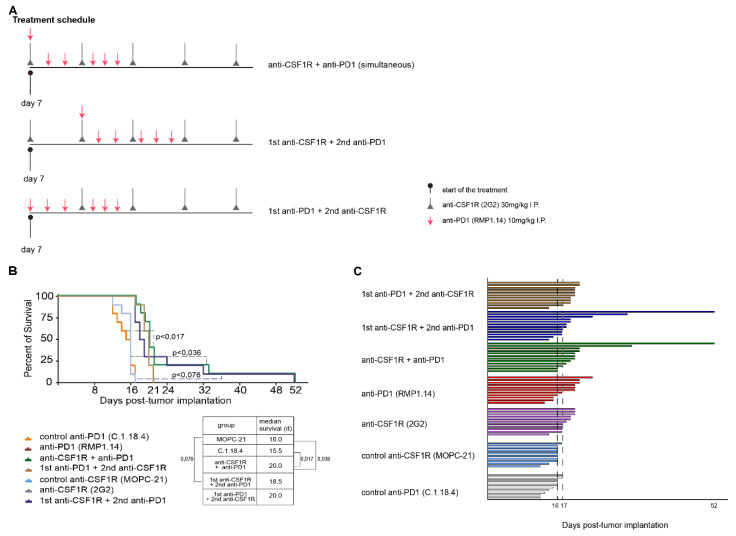
Simultaneous and sequential combinations of PD1 and CSF1R blockade in vivo. (**A**): Schematic overview of experimental design. (**B**): Kaplan–Meier plots: combination therapies vs. monotherapies vs. controls. Experimental groups (*n* = 10 in each group) are as indicated: Blue dashed line shows *p*-value between combination therapy group starting with anti-CSF1R treatment and CSF1R control group. Green dashed lines show *p*-value between both control groups vs. simultaneous combination group. P-values were calculated by using Tukey–Kramer post hoc test after performing Log-rank (Mantel–Cox) test (*p* < 0.0001). (**C**): Symptom-free survival graph displaying each single mouse per experimental group. Experimental groups are: control anti-PD1, control anti-CSF1R, anti-PD1, anti-CSF1R, anti-CSF1R plus anti-PD1, anti-CSF1R and then anti-PD1, and anti-PD1 and then 2nd anti-CSF1R. Dashed line on day 16 represents median latency until experimental endpoint in control group. Dashed line on day 17 shows time point where last animal of control group reached experimental endpoint. Day 52 indicates last surviving animals. Experimental endpoints are described in detail in the material/methods section and in Supplementary Table S1.

Next, we investigated the impact of simultaneous and sequential combination treatments in vivo (as illustrated in Figure 3A). Monotherapies with anti-CSF1R, anti-PD1, and respective control antibodies served as control groups in the experimental setup. The survival times refer to the experimental endpoint as outlined above and in Supplementary Table S1. Median overall survival was prolonged with each monotherapy compared with that of the respective control group (as illustrated in Figure 3B).

Simultaneous treatment with anti-CSF1R and anti-PD1 antibody led to a durable tail of longer-term surviving animals. The sequential treatments only led to longer-term surviving animals when anti-CSF1R antibody was administered before anti-PD1 antibody (as illustrated in Figure 3C). The median overall survival, however, was not significantly prolonged.

### 2.4. Combined Anti-CSF1R and PD1 Antibodies Lead to Decreased CSF1R and Increased CD8/CD4 and CD8/FoxP3 Ratios in Post-Treatment Tissues

**Figure 4:**
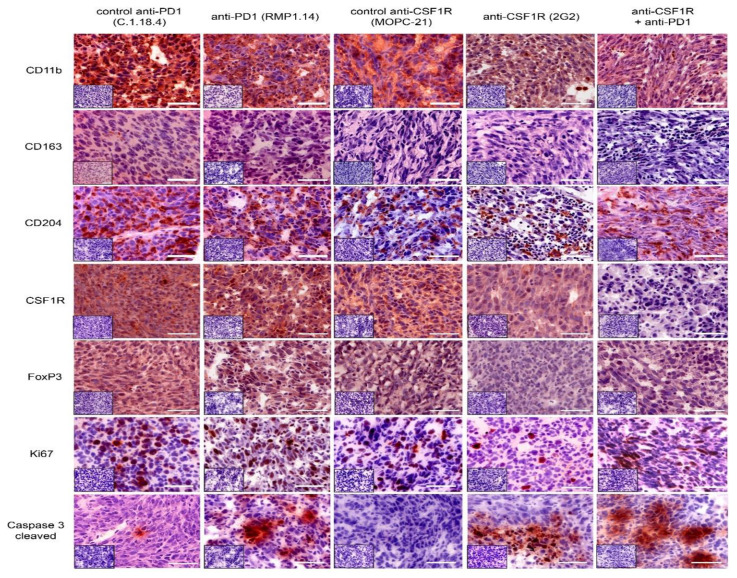
Immunohistochemical analysis in post-treatment tissues (*n* = 3 in each group were analysed). Representative IHC staining patterns of tumor tissues with indicated antibodies after 2 injections of CSF1R antibodies and 3 injections of PD1 antibodies. Small inserts show staining control without application of primary antibody. Scale bars 50 µm.

To further understand the treatment effects of anti-CSF1R and anti-PD1 antibodies, we investigated the immune signature in post-treatment tissues. First, we investigated the post-treatment immune signature after 2 injections of anti-CSF1R and 3 injections of anti-PD1 antibodies, i.e., around day 10 after the onset of treatment. The analysis of macrophage/microglia markers (as illustrated in Figure 4) revealed a reduction of CD11b- and CD204-positive cells with anti-CSF1R antibody monotherapy and combined treatments.

**Figure 5:**
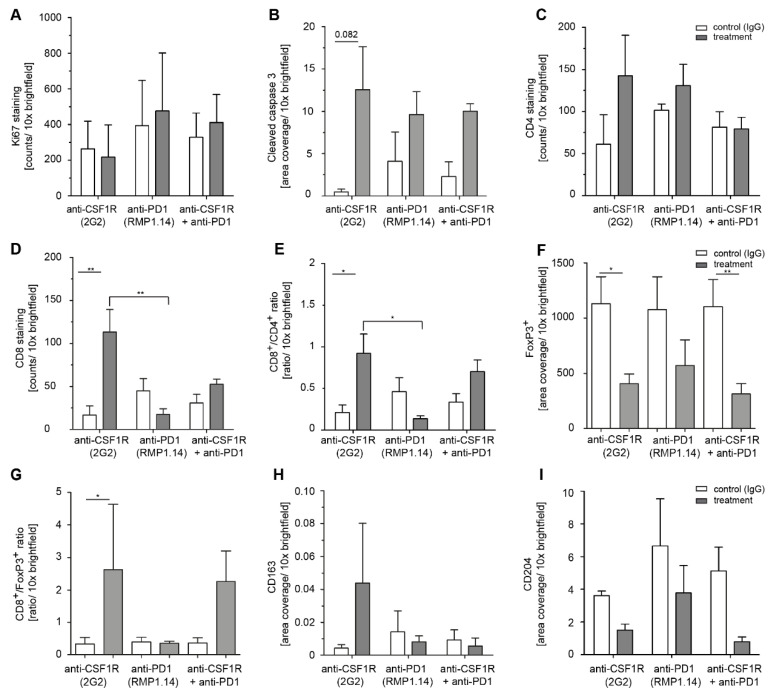
Quantification of Ki67, cleaved caspase 3, CD4, CD8 as well as CD8/CD4, and CD8/FoxP3 ratios in post-treatment tissue. Quantification of Ki67 (**A**), cleaved caspase 3 (**B**), CD4 (**C**), CD8^+^ (**D)**, CD8^+^/CD4^+^ ratio (**E**), FoxP3^+^ (**F**), CD8/FoxP3 ratio (**G**), CD163 (**H),** and CD204 (**I**) in tumor tissues after 2 injections of CSF1R and 3 injections of PD1 antibodies. Three animals (n = 3) in each group were analysed. Statistical analysis was done using one-way ANOVA followed by Tukey’s multiple comparison test. ** *p* < 0.01, * *p* < 0.05.

Of note, the staining intensity of CSF1R was only reduced after anti-CSF1R antibody monotherapy and combined treatments (as illustrated in Figure 4). Ki67 staining did not significantly change after treatments. Treatments with anti-PD1 antibody and anti-CSF1R antibody and their combinations led to a strong signal for cleaved caspase 3. Of note, PD1 and its ligand PD-L1 were present in all treatment groups (as illustrated in Supplementary Figure S4). Quantifications indicated a lack of difference in Ki67 between treatment groups (as illustrated in Figure 5A), an increase of cleaved caspase 3 after anti-CSF1R monotherapy (as illustrated in Figure 5B), and increased CD8^+^/CD4^+^ ratio and CD8^+^/FoxP3^+^ ratio as monotherapy and in combination with anti-PD1 (as illustrated in Figure 5C-F and Supplementary Figure S3). Moreover, quantification of macrophage/microglia marker showed generally weak CD163 staining signal (as illustrated in Figure 5G) and CD204 reduction after anti-CSF1R monotherapy and after combination therapy with an anti-PD1-antibody (as illustrated in Figure 5H).

**Figure 6:**
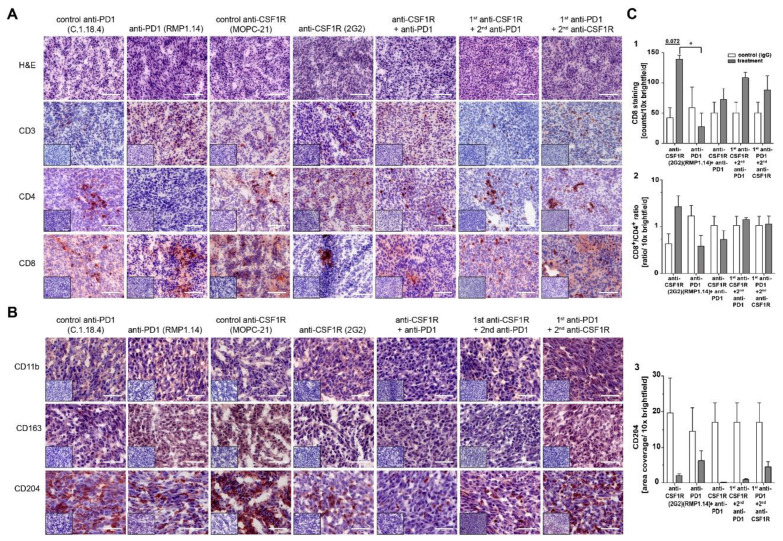
Immunohistochemical analysis of one representative animal per group (*n* = 1) of tumor-infiltrating host cells in simultaneous versus sequential combinations of PD1 and CSF1R blockade in vivo. (**A***)*, H&E and immunohistochemical analysis in representative tumor tissues. Scale bar 100 μm. *(***B***)*, Immunohistochemical analysis in representative tumor tissues. Scale bars 50 μm. Small inserts show staining control without application of primary antibody. (**C**), Quantification of CD8^+^ (**1**), CD8^+^/CD4^+^ ratio (**2**), and CD204^+^ (**3**) cells. For quantification, three tissue samples of different tumor depth per animal were analysed. Statistical analysis was done using one-way ANOVA followed by Tukey’s multiple comparison test. *p* < 0.05.

We performed a thorough immunohistochemical analysis on post-tumor tissues from simultaneous versus sequential treatments involving CD3, CD4, CD8 (as illustrated in Figure 6A), CD11b, CD163, CD204 (as illustrated in Figure 6B), and PD1, PD-L1 (as illustrated in Supplementary Figure S4). Furthermore, we quantified the effects on CD4 (data not shown), CD8, CD8/CD4 ratio, and CD204 (as illustrated in Figure 6C). Anti-CSF1R alone and in combination with anti-PD1 led to 3-fold and 2-fold increased infiltration with CD8^+^ cells (as illustrated in Figure 6C (1,2)), and 6-fold and 1.5-fold higher CD8/CD4 ratio compared with that of respective IgG controls (as illustrated in Figure 6C(3)). The influx of CD4^+^ cells was similar in all treatment groups compared with that of controls (data not shown). CD204^+^ cells decreased after treatments with anti-CSF1R blockade alone (as illustrated in Figure 6B,C). Of note, the reduction of CD204^+^ cells is particularly pronounced after combined treatments of anti-PD1 and anti-CSF1R blockade (as illustrated in Figure 6B, last row, columns 3–5; Figure 6C (3)). We also observed a decrease in CD204^+^ cells after anti-PD1 blockade alone. Yet, this reduction was significantly lower compared to that of the other treatment groups (as illustrated in Figure 6B,C).

### 2.5. Coinhibition of CSF1R and PD1 Enhances Cytotoxicity in Glioblastoma PDM/TILs Co-Cultures Ex Vivo

**Figure 7:**
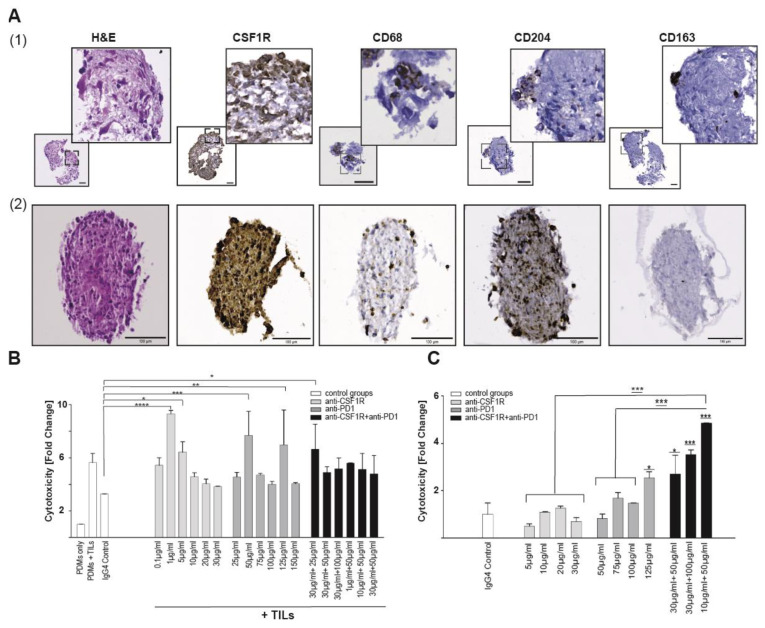
Treatment-induced cytotoxicity in PDMs and PDM/TILs coculture. (**A**) Immunohistochemistry staining of (**1**) PDM model 1 and (**2**) PDM model 2 for markers of macrophages (CD68), tumor-associated macrophages markers (CD204 and CD163), and CSF1R. Scale bars 100 µm. (**B**) PDM model 1, coculture with autologous TILs, treatments and concentrations as indicated after 72 h (*n* = 3 per concentration). Fold changes were normalized to PDMs only. Two-way ANOVA followed by Dunnett’s multiple comparison test was used. PDMs+IgG4-Control served as control group. **** *p* < 0.0001, *** *p* < 0.001, ** *p* < 0.01, * *p* < 0.05. (**C**): PDM model 2 was treated in the absence of TILs with either CSF1R/ PD1 or combination treatments and concentrations as indicated. Cytotoxicity was measured after 72 h. Fold changes were normalized to isotype control; significance above bars refer to control group. Two-way ANOVA followed by Dunnett’s multiple comparison test was used. PDMs +IgG4-Control served as control group. *** *p* < 0.001, * *p* < 0.05.

Based on our results so far, we concluded that anti-CSF1R antibodies reshape the glioma-associated microenvironment by decreasing CD204^+^ cells and increasing the influx of CD8^+^ cells. We aimed at understanding the functional consequence of this observation regarding a combination therapy with anti-PD1 antibody. To this end, we used a patient-derived microtumor (PDM)/ tumor-infiltrating lymphocytes (TILs) coculture model derived from fresh residual tissue of glioblastoma resection by enzymatic tissue digestion (as illustrated in Figure 7B). Isolated autologous TILs were expanded and subsequently used in a coculture experiment with respective PDMs. We further characterized the isolated TILs population by multicolor flow cytometry (as illustrated in Supplementary Figure S5) and detected CD8 and CD4 positive T cells. Further T cell subpopulations widely expressed T cell activation markers like CD107 or CD137 (as illustrated in Supplementary Figure S5).

For monotherapies or combination treatments, we used different concentrations of anti-CSF1R [21] and anti-PD1 antibodies and measured the extent of treatment-induced cytotoxicity (as illustrated in Figure 7C). We did not observe any changes in the treatment-naïve control groups, i.e., PDMs only and PDM + TILs (as illustrated in Figure 7B,C). Anti-CSF1R antibody alone led to increased treatment-induced cytotoxicity at 1 µg and 5 µg/mL. Anti-PD1 antibody alone led to increased treatment-induced cytotoxicity at 50 µg/mL and 125 µg/mL (as illustrated in Figure 7B). Using different concentrations for combination treatments, we observed an increased treatment-induced cytotoxicity already with 25 µg/mL anti-PD1 combined with anti-CSF1R. Immunohistochemical staining of PDM model 1 showed low amounts of infiltrated CD204- and CD163-macrophages together with prominent expression of target protein CSF1R (as illustrated in Figure 7A (1)).

Next, we generated another PDM model (PDM 2) derived from a different tumor sample. Immunohistochemical analysis of PDM model 2 using CSF1R, CD68, CD204, and CD163 revealed the presence of tumor-associated macrophage markers (as illustrated in Figure 7A (2)). Of note, the treatment target CSF1R was strongly present inside PDM 2.

We treated this PDM model 2 without the addition of autologous TILs to investigate the effects of anti-CSF1R and anti-PD1 antibodies on the compartment of tumor-associated macrophages infiltrated into respective PDMs (as illustrated in Figure 7A (2),C).

In contrast to PDM model 1 (as illustrated in Figure 7B), all three tested combination therapy regimes revealed significantly higher cytotoxicity in PDM model 2 (as illustrated in Figure 7C). The most effective treatment regime was the combination of 10µg/mL anti-CSF1R with 50 µg/mL anti-PD1. It showed significantly higher cytotoxicity compared with vehicle and both monotherapies. Monotherapy with anti-PD1 only led to increased cytotoxicity with the highest anti-PD1 concentration (125 µg/mL) (as illustrated in Figure 7C). Yet, by combining CSF1R and anti-PD1, already low concentrations of both compounds led to an increased cytotoxicity in PDM 2. To further validate this result, we investigated the combination therapy in a third PDM model (PDM model 3, as illustrated in Supplementary Figure S6) with positive immunohistochemical CSF1R staining and moderate presence of further TAM markers (as illustrated in Supplementary Figure S6A). Similar to previously tested PDM models, the combination therapy showed highest cytotoxicity in PDM model 3 (as illustrated in Supplementary Figure S6B).

## 3. Discussion

Treatment strategies involving targets in the immunosuppressive glioma-associated microenvironment could be a promising strategy to improve the currently available therapeutic options for glioblastoma patients [23]. Glioma-associated macrophages display distinct tumor-promoting features [24] and contribute to resistance in glioma immunotherapy [25]. In melanoma, for example, macrophage-associated markers including CSF1 were associated with nonresponsiveness to PD1 inhibition [14]. Thus, we investigated anti-CSF1R either alone or in combination with anti-PD1 in experimental glioma. A comprehensive immunophenotyping of newly diagnosed versus progressive glioblastoma investigating tumor-infiltrating leukocytes (TILs) and peripheral blood leukocytes demonstrated an exhaustion signature of TILs in progressive glioblastoma [26]. Of note, this study analysed primary and progressive glioblastoma with matching age-related healthy donors. Immunohistochemical staining in matched paired tumor tissues from primary and corresponding progressive glioblastoma from our center indicated that the relevant targets of the anti-CSF1R and anti-PD1 combination regimen, i.e., CSF1R, the macrophage markers CD204, CD163, and PD1 and PD-L1, were present in patient-derived tissue of newly diagnosed and progressive disease (as illustrated in Figure 1, Supplementary Table S2). Our data confirm previous studies that detected these markers in glioblastoma tissue [17,27]; yet, these studies did not investigate potential treatment-associated alterations between newly diagnosed and progressive disease, nor did they correlate the presence of TILs and TAMs inside the tumor microenvironment. In this context, the performed spearman correlation analysis might suggest that mainly the PD1/PD-L1 axis correlates with histological markers for TAMs (CD204/CD163) and TILs (CD4) in primary and recurrent tissue samples. Our findings might further indicate that TILs infiltration remained comparable in newly diagnosed and corresponding progressive tissue in our cohort (as illustrated in Supplementary Table S3), but larger sample studies will be necessary to validate this finding. A noteworthy observation was that the molecular targets of our compounds, i.e., CSF1R and PD-1, were detected in newly diagnosed and progressive glioblastoma (as illustrated in Figure 1). We conclude that a combined targeting of CSF1R and PD1 in future clinical trials might be feasible in newly diagnosed and as well as in RT/TMZ-treated progressive glioblastoma.

Interestingly, CSF1R blockade alone led to a prolonged latency until the onset of neurological symptoms (as illustrated in Figure 2). As indicated by reduced staining distribution in post-treatment tissues from SMA-560 tumors for CSF1R, CD204, and CD11b after CSF1R monotherapy, the target population is efficiently diminished by the anti-CSF1R antibody in our experimental setup (as illustrated in Figure 2). These results are comparable with other studies combining glioma-associated microenvironment targets with an anti-PD1 checkpoint inhibitor. For example, combinations of C-X-C chemokine receptor type 4 (CXCR-4)/ C-X-C chemokine Ligand (CXCL-12)-axis and led to reduced microglial infiltration and improved PD1 efficacy [28].

We observed that combined treatments with CSF1R and PD1 antibodies altered the immune signature in immunohistochemically analysed post-treatment tissues; in particular, increased T cell infiltration and elevated CD8^+^/CD4^+^ and CD8^+^/FoxP3^+^ ratios (as illustrated in Figure 5 and Figure 6). Higher CD8^+^/CD4^+^ ratios were also observed with anti-CXCR4 and anti-PD1 combination [28]. A recent study on 66 patients [29] highlighted molecular determinants of response to nivolumab. One of the features of nonresponding PTEN-mutant tumors was a markedly reduced immune cell infiltration. Thus, the increased immune cell infiltration by anti-CSF1R observed here might indicate a promising signal for improving the treatment efficacy of PD1 inhibition in glioblastoma. Our data interpretation is further supported by a recent study demonstrating that the combination of anti-PD1 and anti-CSF1R antibodies prolonged the survival of BRAFV600E-driven mouse melanoma [14]. The combination of a CSF1R inhibitor and PD1 reversed the development of immune resistance in a dendritic cell vaccination model [30]. Combinations of PD1 antibodies with inhibition of the T cell exhaustion marker LAG-3 or an inhibition of the tryptophan catabolic enzyme IDO showed comparable results, i.e., increased efficacy of anti-PD1 treatment, later onset of neurological symptoms, and recomposition of the tumor associated microenvironment [31,32]. In our combination treatments, we only observed long-term surviving animals after simultaneous combination treatments or in sequential treatments when PD1 blockade followed CSF1R blockade (as illustrated in Figure 3). This might reflect that the CSF1R blockade-mediated reduction of activated macrophages in post-treatment staining contributes to a better efficacy of subsequent PD1 blockade as indicated by a reduction of CD204+ cells in post-treatment tissues (as illustrated in Figure 2 , Figure 4, Figure 5 and Figure 6). Of note, anti-CSF1R also led to increased influx of CD8^+^ cells (as illustrated in Figure 6). This might further contribute to an efficacy of PD1 blockade. Of course, the limitations of these results need to be considered too; we only observed two long-term surviving mice upon sequential treatments with anti-CSF1R followed by PD1 antibodies. This indicates that further underlying factors determine the efficacy of this combination therapy that need to be investigated in more detail in upcoming studies. Yet, our observations in the PDM culture and PDM/TILs coculture model further support the potential of a combined anti-CSF1R and anti-PD1 strategy: a combined inhibition of PD1 and CSF1R enhanced treatment-induced cytotoxicity (as illustrated in Figure 7B) already at a low concentration of 25 µg/mL of anti-PD1, whereas 25 µg/mL of anti-PD1 monotherapy did not lead to increased treatment-related cytotoxicity (as illustrated in Figure 7B).

Taken together, our study indicates that CSF1R inhibition might be a promising therapeutic strategy for clinical translation in glioblastoma. Furthermore, our data indicate that anti-CSF1R antibody might enhance the efficacy of anti-PD1 antibody even at lower concentrations. Thus, for combinations of anti-CSF1R and anti-PD1, it will be necessary to investigate its sequence and dosage in early phase clinical trials. Recent phase I clinical trials using neoadjuvant dosing of PD1 antibody in progressive glioblastoma suggest that the timing of anti-PD1 antibody needs further consideration [33,34]. Thus, a thorough investigation of novel combinatorial approaches, including anti-CSF1R and anti-PD1, in early phase clinical trials will also have to consider their dosage and timing.

## 4. Materials and Methods

### 4.1. SMA-560 Cell Implantation into Syngeneic VM/Dk Mice

All animal experiments were performed in accordance with the local authorities and the German laws governing the use of experimental animals. All procedures are approved by The Institute of Animal Welfare and the Veterinary Office at the University of Tubingen and the Regional Council Tuebingen. We used the syngeneic SMA-560/VM/Dk mouse model that was described before [35,36,37]).

Five thousand SMA-560 cells were implanted as described previously [38,39]. In brief, adult mice were anesthetized with 3-component anesthesia (fentanyl, midazolam, and medetomidin) before intracranial injection to the right striatum using a fixed stereotactic apparatus. SMA-560 mouse cells were resuspended in 1 × PBS, and 5 × 10^3^ cells in a volume of 2 µL were injected into female or male VM/Dk mice. Glioma-bearing mice were randomized to the experimental groups and were carefully monitored and euthanized at the onset of moderate clinical symptoms, which were evaluated according to a defined scoring system that is outlined in detail in Supplementary Table S1.

### 4.2. Treatment Schedules In Vivo

The CSF1R (2G2), anti-PD1 antibodies and control antibodies (C.1.18.4 and MOPC-21) were provided by Roche Diagnostics (Penzberg, Germany) [21]. Treatments with anti-CSF1R and the control antibody were performed once weekly, 30 mg/kg by intraperitoneal injection. The treatments with anti-PD1 and the control antibody were performed 3 times per week for 2 weeks, 10 mg/kg by intraperitoneal injection.

### 4.3. Scoring of Experimental Animals

After surgery, the animals were closely monitored, and the clinical symptoms were evaluated according to a defined scoring scheme (Supplementary Table S1). The endpoint of the experiments was set at moderate distress. As soon as moderate clinical symptoms were observed, the experimental animals were euthanized conforming to local standards (Regional Council Tuebingen).

### 4.4. Immunohistochemistry of Murine Tumor Samples

The following antibodies were used: CD3, CD4, CD8, CD11b, CD163, FoxP3, Ki67 (Abcam, Cambridge, UK), CD204 (ThermoFisher, Waltham, MA, USA), and cleaved caspase 3 (Cell Signaling, Frankfurt am Main, Germany). Eight µm thick sections were prepared using a Leica CM3050S cryostat and stored at −80 °C. Frozen sections were air-dried at room temperature for 10 min, fixed in ice-cold acetone at −20 °C for 10 min or 4% PFA for 15 min. Bloxall (Vector Laboratories, Peterborough, UK) was used to quench endogenous peroxidase activity. Slides were incubated with 10% bovine serum albumin (BSA) in PBS-Tween 0.3% for 1 h at room temperature and then incubated with primary antibody in a humidity chamber overnight at 4 °C. The following day, slides were incubated for 1 h at room temperature with biotinylated secondary antibodies, and positive staining was detected using Vector NovaRED (Vector Laboratories, Burlingame, CA, USA). Stained tissue sections were investigated under Carl Zeiss Axioplan2 Imaging brightfield microscope. Staining analyses and picture processing were performed using Fiji ImageJ (National Institutes of Health, Bethesda, MA, USA).

### 4.5. Immunohistochemistry of Human Glioblastoma Samples

We obtained the approval by the ethical board of the University Hospital Tübingen (permission number 077/2016BO2). We identified 34 patients who were treated at our Neuro-oncology Centre where samples were available from the newly diagnosed treatment-naïve tissue and from first progression. All samples were classified as glioblastoma, IDH-wildtype, WHO grade IV according to the current WHO classification of central nervous system (CNS) tumors. Formalin-fixed, paraffin-embedded tissue microarray sections were stained for CD3 (1:500, 40 min CC1 pretreatment, clone SP7, ThermoFisher, Waltham, MA, USA), CD4 (1:2, 24 min CC1, clone SP35, Ventana Medical Systems, Roche Group, Indianapolis, IN, USA), CD8 (RTU, 64 min CC1, clone SP57, Ventana Medical Systems, Roche Group, USA), CD163 (RTU, MRQ-26, Ventana Medical Systems, Roche Group, USA), CSF1R (dilution 1:2500, 32 min CC1, clone 29, Roche Diagnostics GmbH, Penzberg, Germany), PD1 (1:100, 64 min CC2, Clone MRQ-22, Zytomed, Berlin, Germany), PD-L1 (1:100, 64 min CC1, ab205921, Abcam, Cambridge, UK), and CD204 (1:2500, 32 min CC1, HPA000272, Sigma Aldrich, St. Louis, MI, USA) on the Ventana Benchmark XT. immunohistochemistry system with a 32 min antibody incubation time each. The slides were scanned at 20x using either the Ventana iScan HT or the Hamamatsu Nanozoomer^®^ bright field scanner, and positively stained cells within tumor tissue were evaluated and quantified manually or by a semiautomated staining quantification using ImageJ (National Institutes of Health, Bethesda, MA, USA, https://imagej.nih.gov/ij1997–2018/, (accessed on 1 September.2020) as follows: none (<1% positive cells), low (≤25%), intermediate (≤50%), high (≤75%), very high (>75%).

Expression levels (as outlined in Supplementary Table S2) represent stained area percentage of whole tissue cores and were evaluated either manually or by a semiautomated staining quantification using ImageJ. Expression levels were grouped in 4 or 5 interval-based subgroups, groups were represented by values 0 to 4, and mean values were calculated. Additionally, an established immunoreactive score (IRS) was generated (as shown in Supplementary Figure S1). [40,41] The staining intensity of tissue samples was primarily semi-quantitatively scored as 0 (absent staining signal), 1 (weak expression), 2 (moderate expression), and 3 (strong expression). IRS was formed by multiplication of the intensity score and semiquantitative staining quantification (score 0–4, as outlined above). Difference in sample numbers is caused by incomplete transfer of tissue cores on the tissue microarray and number of matched pair sample sets.

### 4.6. Patient-Derived Microtumors (PDMs) and Tumor Infiltrating Lymphocytes (TILs)

We used fresh residual tumor tissue from glioblastoma resections and generated microtumors. The ethical board of the University Hospital Tübingen approved this study. We kept tumor tissue in DMEM F12 (Sigma Aldrich, St. Louis, MO, USA) plus 1% Primocin (Invivogen, San Diego, CA, USA), and washed samples with Hank’s Balanced Salt Solution (HBSS; Thermo Fisher, Waltham, MA, USA). Tissue fragments were crushed into small (1–2 mm) pieces and were washed again. A digestion step was performed using a medium containing 0.28 U/mL Liberase DH (Sigma Aldrich, St. Louis, MO, USA) solution and incubated at 37 °C. Afterwards, the medium was discarded, and samples were washed and sequentially filtered through a stainless-steel wire mesh (500 µm hole size; Fisher Scientific, Waltham, MA, USA) and a 40 µm cell strainer (pluri Select Life Science, Leipzig, Germany). For TILs isolation, single cells of the flow-through were collected and stored in liquid nitrogen.

PDMs were carefully collected and cultured in StemPro^®^ hESC SFM medium (Thermo Fisher, Waltham, MA, USA) with bFGF (10 µg/mL; Peprotech, Rocky Hill, NJ, USA) and 1% Primocin (Invivogen, San Diego, CA, USA) at 5% CO_2_ and 37 °C. Isolated cells of the flow-through were resuspended for TILs expansion in T cell medium (Advanced RPMI (Sigma Aldrich, St. Louis, MO, USA), containing Glutamine (200 mM; Thermo Fisher, Waltham, MA, USA), 1× MEM Vitamins (Thermo Fisher, Waltham, MA, USA), human AB serum (5%; Sigma Aldrich, St. Louis, MO, USA), Primocin (1%; Invivogen, San Diego, CA, USA) containing IL-15 (23.8 U/mL; Peprotech, Rocky Hill, NJ, USA), IL-2 (100 U/mL; Peprotech, Rocky Hill, NJ, USA), IL-7 (10U/mL, Peprotech, Rocky Hill, NJ, USA), and CD3-/CD28-coated magnetic beads (Dynabeads Human T-Activator CD3/CD28, Thermo Fisher, Waltham, MA, USA). TILs were expanded at 5% CO_2_ and 37 °C.

PDM viability was assessed by costaining with Calcein-AM (Thermo Fisher Scientific; green channel) for highlighting viable cells and SyTOX Orange (Thermo Fisher Scientific; red channel) for identification of dead cells.

### 4.7. Flow Cytometry for the Characterization of PDM-Derived TILs

We used FIX&PERM Cell Permeabilization Kit (Thermo Fisher, Waltham, MA, USA) for fixation and permeabilization. TILs immune phenotypes were analyzed on an LSR Fortessa cytometer (Beckton, Dickinson & Company, Franklin Lakes, NJ, USA) using the following antibodies: Anti-CD4-BV510, Anti-CD107a-BV605, Anti-CD8-PerCP/Cy5.5, Anti-CD3-FITC, Anti-CD137-APC/Cy7, and Anti-CD25-Alexa Fluor 700 (all antibodies purchased from BioLegend, San Diego, CA, USA). Data analysis was performed using FlowJo v10.6.2.

### 4.8. Coculture Cytotoxicity Assay

PDMs were cultured in 96-well plates together with autologous TILs at an effector: target cell ratio of 4:1 with the CellTox™ Green Cytotoxicity Assay reagent (Promega, Madison, WI, USA) [42]. Treatments included anti-CSF1R antibody [21], anti-PD1 antibody (Absource Diagnostics GmbH, Munich, Germany), and the respective human IgG4 isotype control (Invivogen, San Diego, CA, USA) at indicated concentrations and time points (each measured in triplicates). Fluorescence assay signal was measured using a multimode microplate reader (Excitation filter: 485 (20) nm, Emission filter: 535 (20) nm; Tecan, Männedorf, Switzerland). Measured fluorescence units were background corrected and plotted, and the resulting fold change values normalized to isotype treated controls.

### 4.9. Immunohistochemistry of PDMs

PDMs were isolated as described above (4.6), collected using 40 µm cell strainers (Corning, Glendale, AZ, USA), washed twice in HBSS (Thermo Fisher Scientific), and fixed in 4% phosphate-buffered formaldehyde solution at pH7 (Carl Roth, Karlsruhe, Germany) for 1 h at room temperature. Next, PDMs were stained with hematoxylin (Leica Biosystems, Nußloch, Germany) for 5 min, washed briefly in H_2_O, and incubated twice in 50% EtOH and 70% EtOH for 15 min each. PDMs were then embedded into a gel matrix (Richard–Allan Scientific HistoGel, Fisher Scientific, Waltham, MA, USA) using a cryomold (Sakura Finetek, Staufen im Breisgau, Germany) according to manufacturer’s instructions. The gel matrix containing PDMs was stored in 70% EtOH for up to 2 weeks until further processing for immunohistochemistry. For immunohistochemistry analyses, gel-embedded PDMs were embedded into paraffin blocks. 5 µm sections were subjected to H&E staining (Leica Biosystems) as well as IHC staining using a DAB (3,3′-Diaminobenzidine) staining solution (Leica Biosystems). The following antibodies were used for IHC staining of PDM sections: CSF1R (used at 1:200 dilution; Catalog Number: 25949-1-AP, Proteintech, Manchester, UK), MSR1/CD204 (used at 1:1000 dilution; Catalog Number: HPA000272, Atlas Antibodies AB, Bromma, Sweden), CD68 (used at 1:400 dilution, clone D4B9C, Catalog number: 76,437, Cell Signaling Technology, Danvers, MA, USA), and CD163 (used at 1:500 dilution, clone D6U1J, Catalog number: 93,498, Cell Signaling Technology). Stained sections were imaged on an Axio Scan.Z1 Slide Scanner (Carl Zeiss, Oberkochen, Germany) and equipped with an EC Plan-Neofluar 20×/0.5 objective (Carl Zeiss) and a Hitachi HV-F203SCL CCD color camera (Hitachi, Tokyo, Japan).

### 4.10. Statistics

*P* values of IHC quantification were generated by using one-way Analysis of variance (ANOVA) with Tukey’s multiple comparison test (GraphPad Prism 9). In in vivo survival studies, Kaplan–Meier method (Kaplan–Meier survival fractions) was used to generate *p* values and calculate the Log–rank (Mantel–Cox). Moreover, the Tukey–Kramer post hoc test was used. Error bars represent standard error of the mean (SEM). For the analysis of the immunohistochemical staining, a correlation of tissue-dependent markers was assessed using spearman’s rank correlation. Correlation coefficient r was calculated and results showing r > 0.30 with related *p*-values were included in Supplementary Figure S2. Effect sizes were interpreted referring to Cohen’s standard, which describes r ≥ 0.1 as small association, r ≥ 0.3 as moderate association, and r ≥ 0.5 as strong association [43]. Statistical significance in the coculture experiment was primarily tested with a two-way ANOVA test followed by an Dunnett multiple comparison test (GraphPad Prism 8).

## 5. Conclusions

In summary, we report here data for a targeting of anti-CSF1R alone or in combination with anti-PD1 in vivo and ex vivo. We conclude that our data contribute a novel therapeutic strategy for clinical translation in future early phase clinical trials for glioblastoma patients.

## Data Availability

The data presented in this study are available in this article.

## Supplementary Materials

The following are available online at https://www.mdpi.com/article/10.3390/cancers13102400/s1, Figure S1: frequency of alternative quantification of human brain tissue samples by a calculated immunoreactive score (IRS); Figure S2: scatter plots outlining statistical correlation analysis of immunohistochemical markers in tissue samples from newly diagnosed and corresponding progressive glioblastoma; Figure S3: immunohistochemical analysis in post treatment tissue (CD3, CD4, and CD8); Figure S4: immunohistochemical analysis of PD1 and PD-L1; Figure S5: PDM morphology and TILs characterization of PDM model 1; Figure S6: treatment-induced cytotoxicity in PDM model 3; Table S1: parameter for scoring of the experimental animals; Table S2: semiquantitative analysis of immunohistochemical staining; Table S3: expression changes between primary and recurrent tumor tissue samples.
