# Supplementary material for: Targeting CSF1R Alone or in Combination with PD1 in Experimental Glioma
Source: Cancers (Basel). 2021 May 15;13(10):2400. doi: 10.3390/cancers13102400 (PMC8156558; doi:10.3390/cancers13102400)
Supplement: Supplementary file 1 [file cancers-13-02400-s001.zip › cancers-1148904-SI.pdf]

cancers

MDPI

Supplementary information

Targeting CSF1R Alone or in Combination with PD1 in Experimental Glioma

Justyna M. Przystal, Hannes Becker, Denis Canjuga, Foteini Tsiami, Nicole Anderle, Anna-Lena Keller, Anja Pohl, Carola H. Ries, Martina Schmittnaegel, Nataliya Korinetska, Marilin Koch, Jens Schittenhelm, Marcos Tatagiba, Christian Schmees, Susanne C. Beck and Ghazaleh Tabatabai

Table S1. Parameter for scoring of the experimental animals.

Parameter	Phenotype	Score
**General appearance**	Clean skin and orifices, no pain, no weight loss	0
	Slight eye or nose discharge, slight pain, up to 10% weight loss	1
	Sticky eyes, moderate pain, up to 19% weight loss	2
	Cramps, dehydration, strong pain, max. 20% weight loss	3
**Behavior and motion activity**	Normal spontaneous-explorative behavior, normal activity	0
	Reduced spontaneous-explorative behavior, reduced activity	1
	Strongly reduced spontaneous-explorative behavior, strongly reduced activity	2
	Total inactivity	3
**Posture, facial expression and assessment of pain with the "grimace score" (1)**	Normal posture, normal facial expression	0
	Slightly hunched back, less than 5 facial attributes with score 1, little pain	1
	Moderately hunched back, grimace score: all facial attributes are moderate, moderate pain	2
	Strongly hunched back, grimace score: all facial attributes are severe, severe pain	3
**Neurological symptoms** (Behaviour in the cage and on the grid, left paw paralysis)	None	0
	Slight loss-of-balance, occasionally missed steps, slight paralysis	1
	Moderate loss-of-balance, every third step missed, moderate paralysis	2
	Strong loss-of-balance, total inactivity, strong paralysis	3

Table S2. Semi-quantitative analysis of immunohistochemical stainings.

	Newly Diagnosed Glioblastoma		Progressive Glioblastoma	
	**N**	**%**	**n**	**%**
CSF1R expression	n=28			
none (<1%)	2/28	7.1	2/28	7.1
low (≤ 10%)	12/28	42.9	15/28	53.6
intermediate (≤25%)	6/28	21.4	5/28	17.9
high (> 25%)	8/28	28.6	6/28	21.4
Mean value	1.71		1.54	
CD204 expression	n= 27			
none (<1%)	0/27	0	0/27	0
low (≤25%)	4/27	11.1	3/27	11.1
intermediate (≤50%)	9/27	33.3	9/27	37.3
high (≤75%)	8/27	33.3	8/27	25.9
very high (>75%)	6/27	22.2	7/27	25.9

Mean value	2.67		2.67	
CD163 expression	n=31			
none (<1%)	8/31	25.8	2/31	3.2
low (≤25%)	9/31	29.0	17/31	58.0
intermediate (≤50%)	7/31	22.6	9/31	29.0
high (≤75%)	7/31	22.6	3/31	9.7
very high (>75%)	0/31	0	0/31	0
Mean value	1.42		1.45	
PD1 expression	n=30			
none (<1%)	20/30	66.7	22/30	76.7
low (≤25%)	9/30	30.0	7/30	23.3
intermediate (≤50%)	1/30	3.3	1/30	0
high (≤75%)	0/30	0	0/30	0
very high (>75%)	0/30	0	0/30	0
Mean value	0.37		0.23	
PD-L1 expression	n=31			
none (<1%)	5/31	16.1	1/31	3.2
low (≤25%)	16/31	51.6	17/31	54.8
intermediate (≤50%)	7/31	22.6	11/31	35.5
high (≤75%)	3/31	9.7	2/31	6.5
very high (>75%)	0/31	0	0/31	0
Mean value	1.26		1.45	
CD3expression	n=28			
none (<1%)	4/28	14.3	4/28	14.3
low (≤5%)	17/28	60.7	15/28	57.1
intermediate (≤10%)	6/28	25.0	9/28	28.6
high (≤15%)	1/28	0	0/28	0
very high (>15%)	0/28	0	0/28	0
Mean value	1.11		1.14	
CD4 expression	n=30			
none (<1%)	3/30	10.0	1/30	3.3
low (≤5%)	5/30	16.7	3/30	10.0
intermediate (≤10%)	9/30	30.0	10/30	33.3
high (≤15%)	6/30	20.0	7/30	23.3
very high (>15%)	7/30	23.3	9/30	30.0
Mean value	2.3		2.67	
CD8 expression	n=28			
none (<1%)	5/28	17.9	2/28	7.1
low (≤5%)	17/28	60.7	20/28	71.4
intermediate (≤10%)	6/28	21.4	5/28	17.9
high (≤15%)	0/28	0	1/28	3.6
very high (>15%)	0/28	0	0/28	0
Mean value	1.04		1.21	

Table S3. Expression changes between primary and recurrent tumor tissue samples.

**Marker**	**n**	**Primary > Recurrent**		**Recurrent> Primary**		**Equal Expression**	
		**N**	**%**	**n**	**%**	**n**	**%**
CSF1R	28	7	25	5	17.8	16	57.1
CD204	27	7	25.6	7	25.6	13	48.1
CD163	30	9	30.0	12	40.0	9	30.0
PD1	30	7	23.3	4	13.3	19	63.3
PD-L1	31	5	16.1	11	35.5	15	48.4

CD3	28	8	28.6	7	25.0	13	46.4
CD4	30	10	33.3	6	20.0	14	46.6
CD8	28	9	32.1	5	17.9	14	50

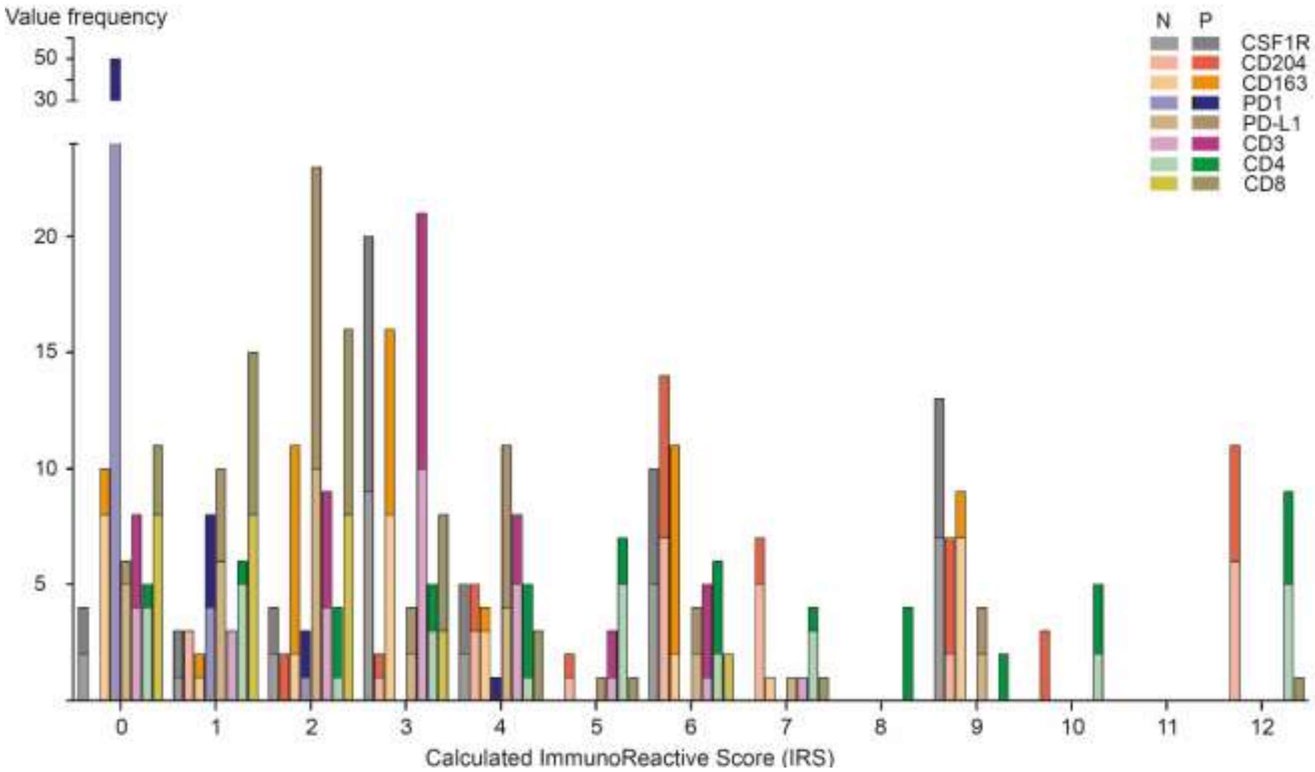

Figure S1. Frequency of alternative quantification of human tissue samples by a calculated immunoreactive score (IRS). IRS was obtained by multiplying staining intensity score with semi-quantitative score of Supplementary table 2 (range 0-12). Frequency of values are shown above. Sample numbers are as indicated in Supplementary table 2. Legend: "N": newly diagnosed Glioblastoma. "P": Progressive Glioblastoma.

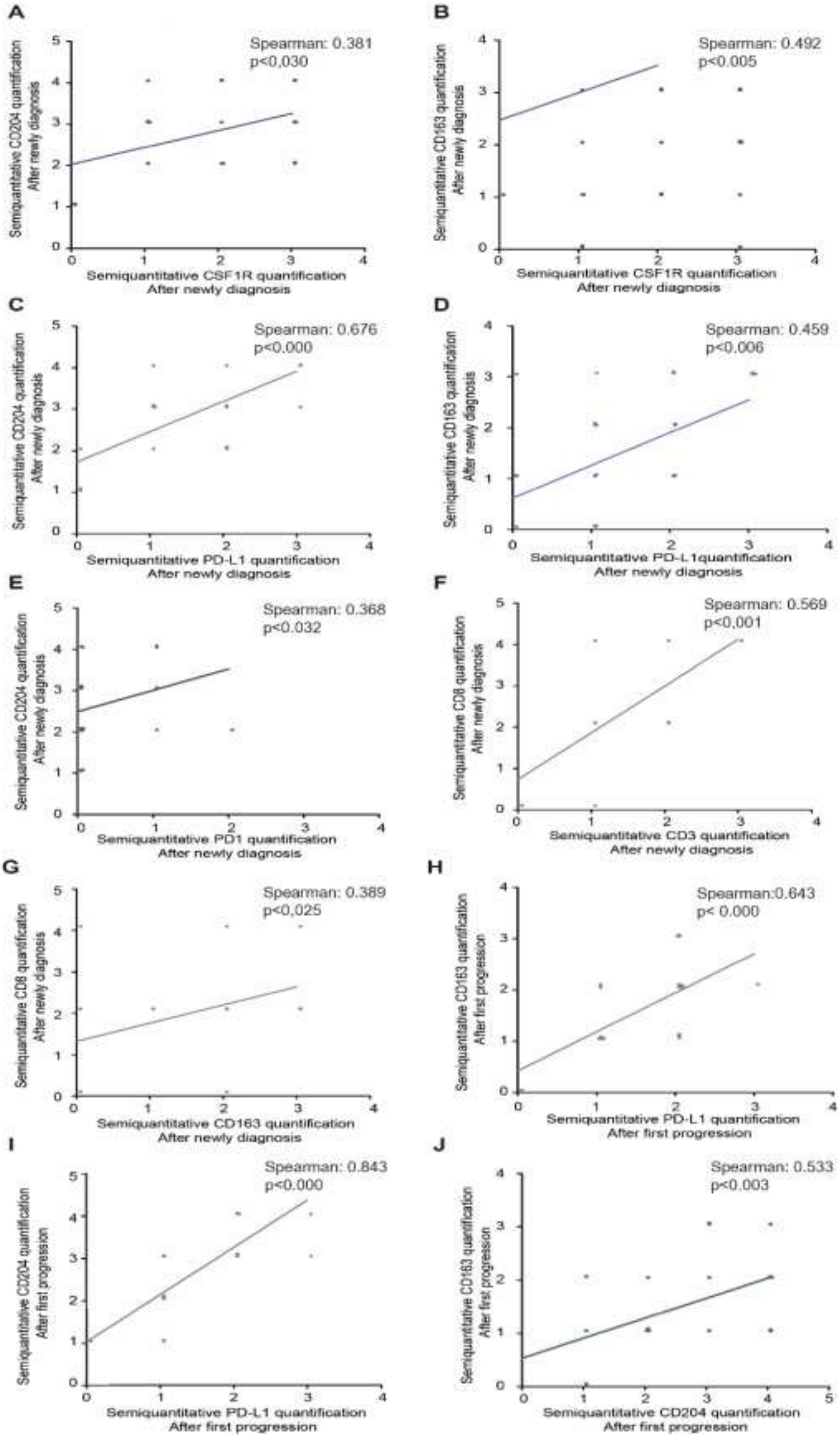

Figure S2. Scatter plots outlining statistical correlation analysis of immunohistochemical markers in tissue samples from newly diagnosed and corresponding progressive glioblastoma. Semiquantitiative quantification of CSF-1R and CD204 (A), CSF1R and CD163 (B), PD-L1 and CD204 (C), PD-L1

and CD163 (D), PD1 and CD204 (E), CD3 and CD8 (F), CD163 and CD8 (G) in newly diagnosed glioblastoma. Semiquantitative quantification of PD-L1 and CD163 (H), PD-L1 and CD204 (I), CD204 and CD163 (J) at first progression of glioblastoma. The spearman correlation test was used for this analysis. Spearman correlation coefficient r and additional p-values as indicated. Sample numbers are as indicated in Supplementary table 2.

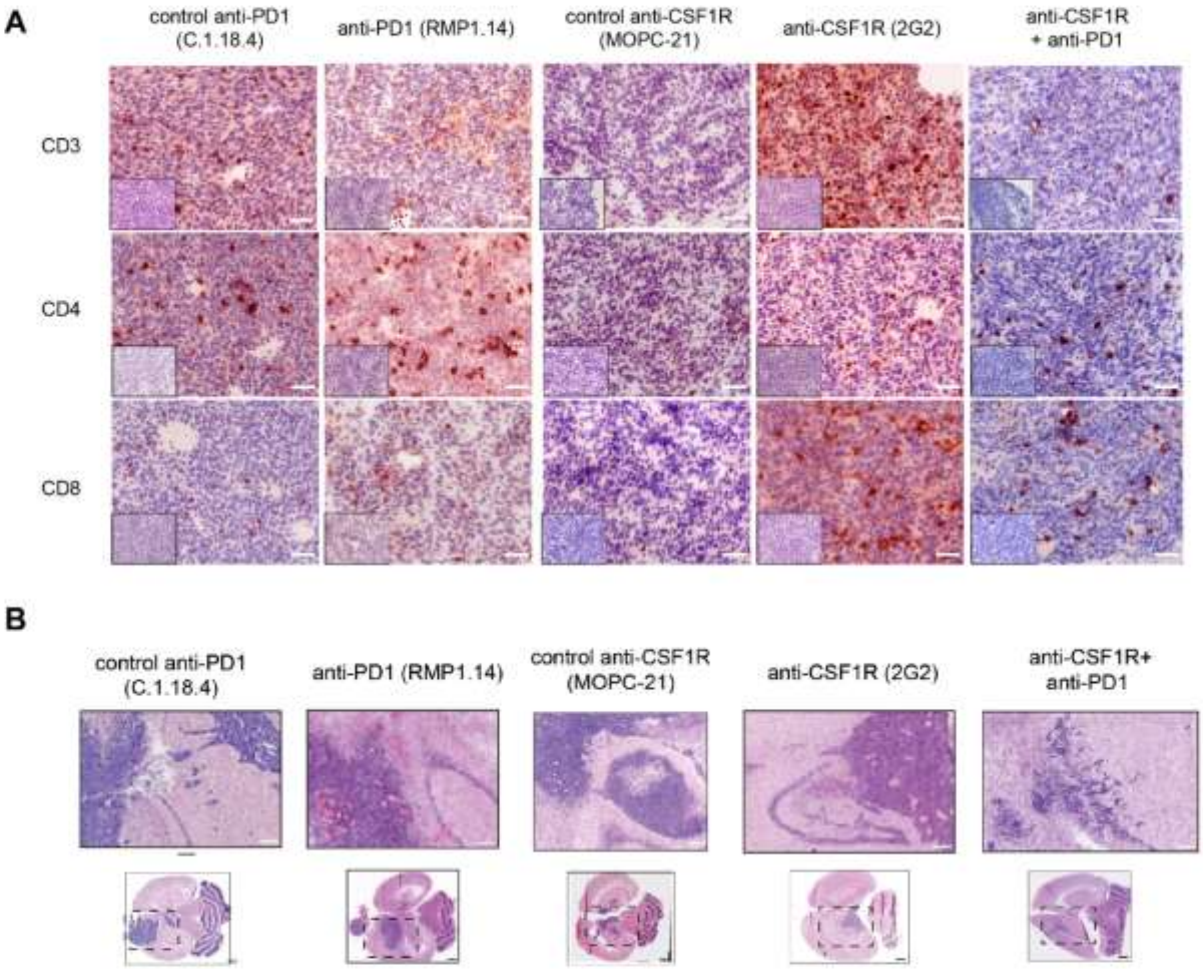

Sup. Fig.3

Figure S3. (**A**) Immunohistochemical analysis in post-treatment tissues (n=3 in each group were an-alysed). Immunohistochemical analysis in representative tumor tissues with the indicated antibod-ies after 2 injections of CSF1R antibodies and 3 injections of PD1 antibodies (as in Figure 4). Small inserts show staining control without application of primary antibody. Scale bars 50 µm. (**B**) Whole brain HE sections illustrating the infiltrative growth of the SMA560 model. Sections of exemplary animals, of the respective treatment group as indicated above. Upper image, 5x magnification, scale bar 200µm. Bottom: overview image, scale bar 1000µm.

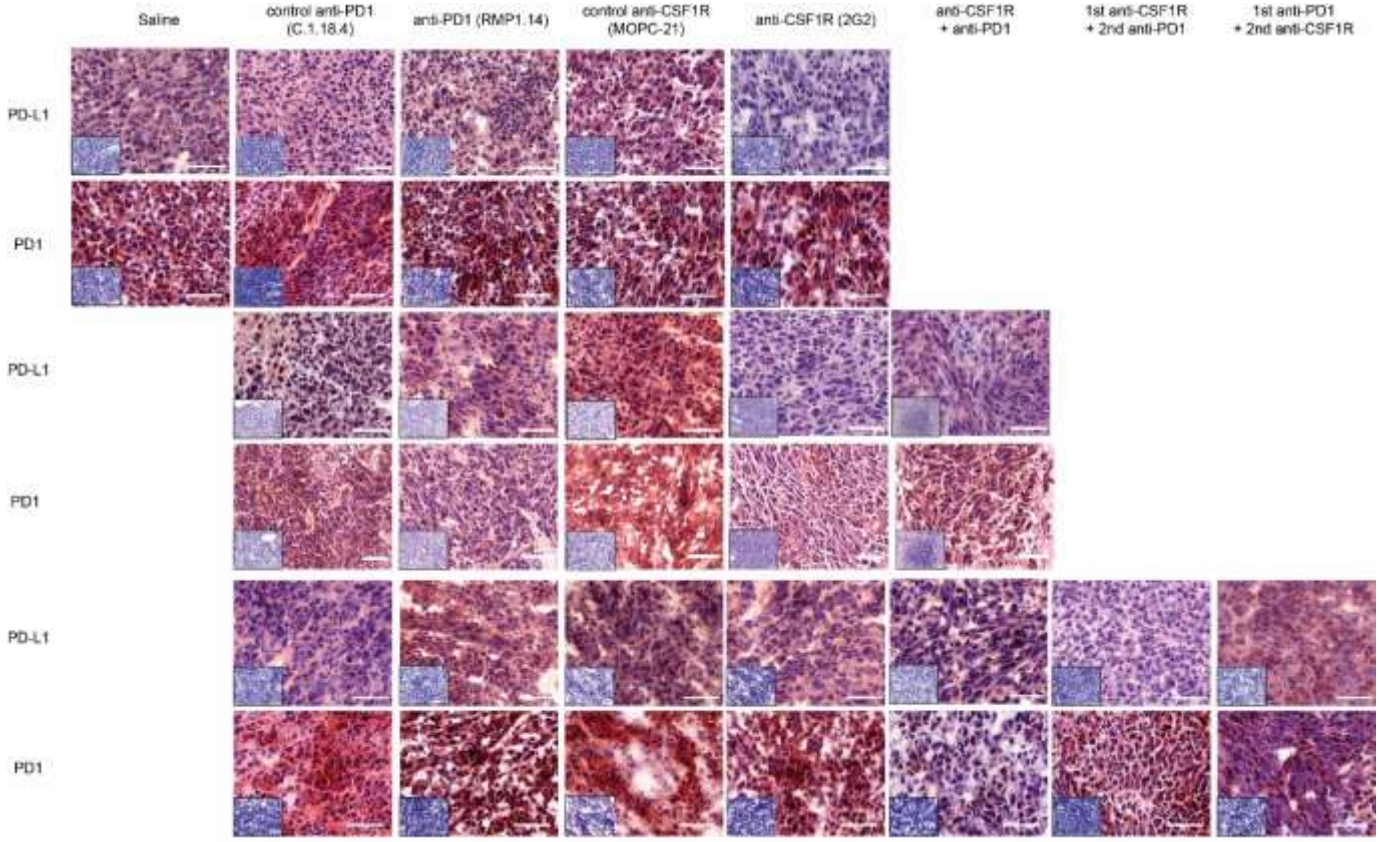

Figure S4. Immunohistochemical analysis of PD1 and PD-L1 expression in post-treatment tissues (in Figures 2, 5 and 6 in the main manuscript). Row 1 and 2, PD-L1 and PD1 expression refer to Figure 2; rows 3 and 4 refer to Figure 4; rows 5 and 6 row refer to Figure 6 (further details are outlined in the text). One animal (n=1) per group was analysed. Small inserts show staining control without application of primary antibody Scale bars 50 µm.

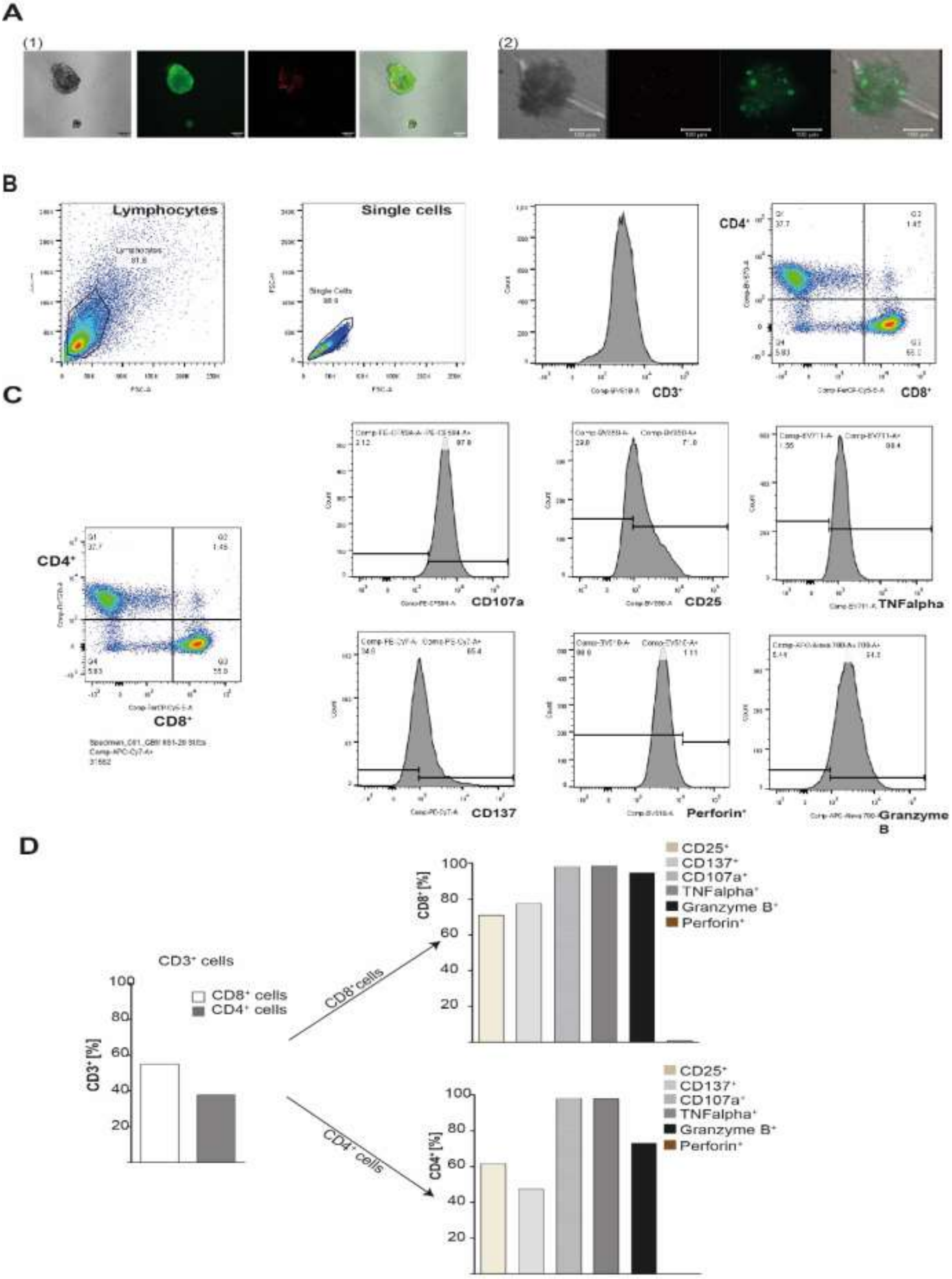
A
(1)
(2)
B
Lymphocytes
Single cells
CD4⁺
CD3⁺
CD8⁺
C
CD4⁺
CD8⁺
CD107a
CD25
TNFalpha
CD137
Perforin⁺
Granzyme B
D
CD3⁺ cells
CD8⁺ cells
CD4⁺ cells
CD3⁺ [%]
CD8⁺ cells
CD4⁺ cells
CD8⁺ [%]
CD4⁺ [%]
CD25⁺
CD137⁺
CD107a⁺
TNFalpha⁺
Granzyme B⁺
Perforin⁺
100
80
60
40
20

Figure S5. PDM morphology and TIL characterization of PDM model 1. (**A**) Representative fluorescent pictures highlighting viability of PDM model (1) 1 and (2) 2 following live-dead cell staining with calcein-AM (green channel, viable cells) and SyTOX Orange (red channel; dead cells). Scale bars 200μm. (**B**) T cell gating strategy using multi-color flow cytometry. Exemplary workflow for separating CD4+ and CD8+ cell population is shown. (**C**) Subpopulations of CD4+ and CD8+ cells are gated for T cell activation markers CD25, CD107a, CD137, Granzyme B and TNFα. (**D**) Quantification of T cell activation markers, characterizing TIL fraction of PDM model 1.

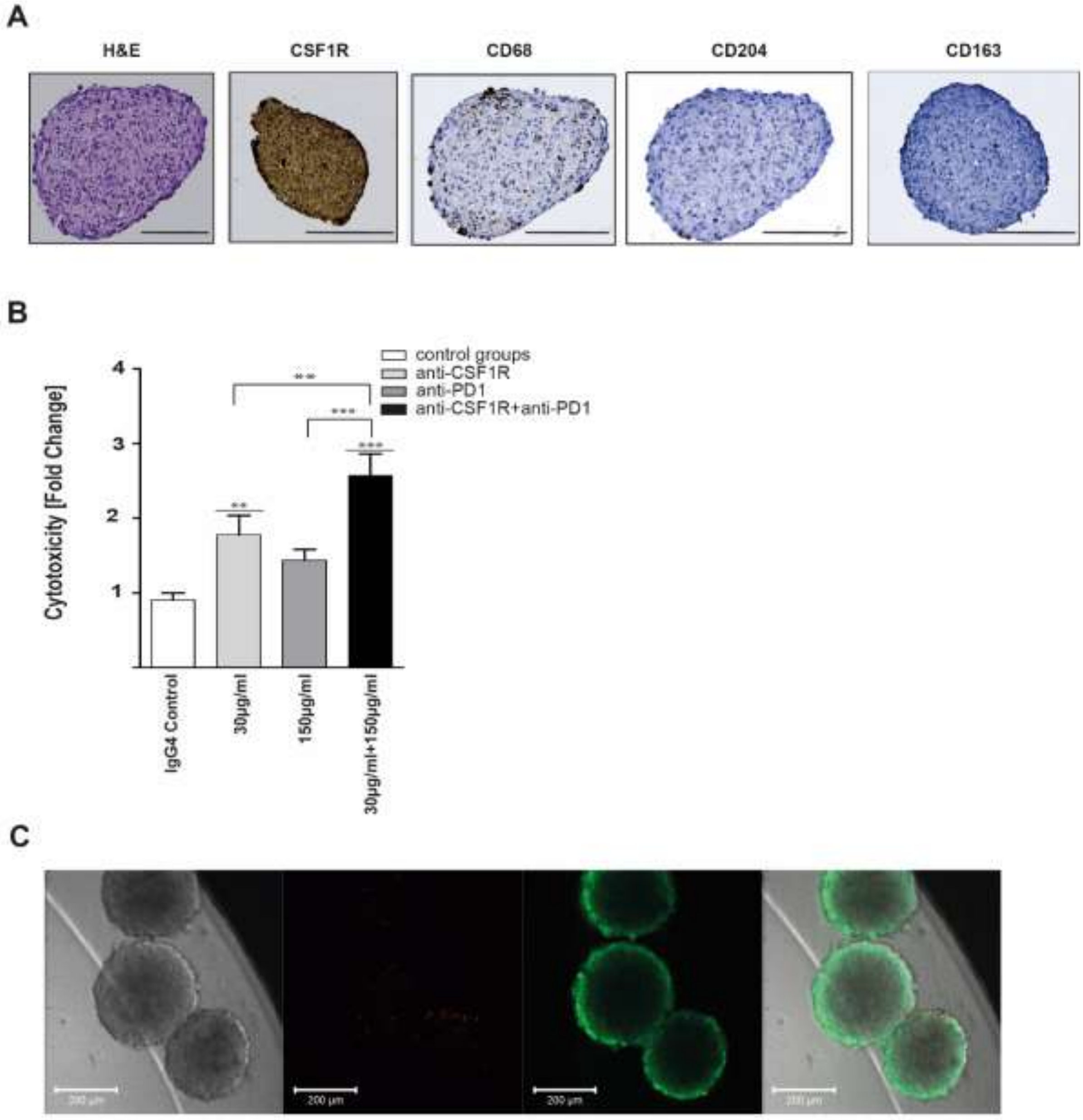

Figure S6. Treatment-induced cytotoxicity in PDM Model 3. (A) Immunohistochemistry staining of PDM model 3 for markers of macrophages (CD68), and tumor-associated macrophages (CD204 and CD163) as well as CSF1R. Scale bars 100μm. (B) PDM model 3 was treated in the absence of TILs with either CSF1R/ PD1 or combination, treatments and concentrations as indicated. Cytotoxicity was measured after 72h. Fold changes were normalized to isotype control, significance above bars refer to control group. Two-way ANOVA followed by Dunnett's multiple comparison test was used.

PDMs +IgG4-Control served as control group. ***P<0.001 and **P<0.01. (C) Representative fluorescent pictures of PDM model 3. Live dead cell staining with calcein-AM (green channel, viable cells) and SyTOX Orange (red channel; dead cells). Scale bars 200 μm.

Reference

1. Langford, D.J.; Bailey, A.L.; Chanda, M.L.; Clarke, S.E.; Drummond, T.E.; Echols, S.; Glick, S.; Ingrao, J.; Klassen-Ross, T.; Lacroix-Fralish, M.L.; et al. Coding of facial expressions of pain in the laboratory mouse. *Nat. Methods* **2010**, 7, 447–449.
